# Nicotianamine-chelated iron positively affects iron status, intestinal morphology and microbial populations *in vivo* (*Gallus gallus*)

**DOI:** 10.1038/s41598-020-57598-3

**Published:** 2020-02-10

**Authors:** Jesse T. Beasley, Alexander A. T. Johnson, Nikolai Kolba, Julien P. Bonneau, Raymond P. Glahn, Lital Ozeri, Omry Koren, Elad Tako

**Affiliations:** 1 School of BioSciences, The University of Melbourne, Victoria, 3010 Australia; 2 Robert W. Holley Center for Agriculture and Health, USDA-ARS, Ithaca, New York 14853 USA; 3 Azrieli Faculty of Medicine, Bar-Ilan University, Safed, 1311502 Israel

**Keywords:** Plant sciences, Physiology

## Abstract

Wheat flour iron (Fe) fortification is mandatory in 75 countries worldwide yet many Fe fortificants, such as Fe-ethylenediaminetetraacetate (EDTA), result in unwanted sensory properties and/or gastrointestinal dysfunction and dysbiosis. Nicotianamine (NA) is a natural chelator of Fe, zinc (Zn) and other metals in higher plants and NA-chelated Fe is highly bioavailable *in vitro*. In graminaceous plants NA serves as the biosynthetic precursor to 2′ -deoxymugineic acid (DMA), a related Fe chelator and enhancer of Fe bioavailability, and increased NA/DMA biosynthesis has proved an effective Fe biofortification strategy in several cereal crops. Here we utilized the chicken (*Gallus gallus*) model to investigate impacts of NA-chelated Fe on Fe status and gastrointestinal health when delivered to chickens through intraamniotic administration (short-term exposure) or over a period of six weeks as part of a biofortified wheat diet containing increased NA, Fe, Zn and DMA (long-term exposure). Striking similarities in host Fe status, intestinal functionality and gut microbiome were observed between the short-term and long-term treatments, suggesting that the effects were largely if not entirely due to consumption of NA-chelated Fe. These results provide strong support for wheat with increased NA-chelated Fe as an effective biofortification strategy and uncover novel impacts of NA-chelated Fe on gastrointestinal health and functionality.

## Introduction

Iron (Fe) supplementation and fortification are the two most widely used strategies to combat human Fe deficiencies that affect over 2 billion people worldwide^1–3^. Iron supplementation involves large dose delivery of highly absorbable (bioavailable) Fe to humans and is effective in treating severe cases of Fe deficiency anemia^2,4,5^. Iron fortification involves low dose delivery of bioavailable Fe fortificants to food products during manufacture (or point-of-use) and is an effective population-based strategy to boost Fe intakes. Iron fortification of wheat flour is now mandatory in 75 countries worldwide (Flour Fortification Initiative; https://fortificationdata.org/), however, the tendency of Fe fortificants such as ferrous sulfate (FeSO_4_) to oxidize and cause undesired organoleptic and sensory properties pose significant challenges^6,7^. Almost 90% of countries utilize fortificants with poor bioavailability or fortify at sub-optimal concentrations, although recent evidence suggests that Fe fortification can effectively reduce symptoms of Fe-deficiency anemia when correctly implemented^8–10^. Iron chelated by ethylenediaminetetraacetate (EDTA) is a commonly recommended fortificant for cereal flour to minimize sensory alterations while providing Fe in a bioavailable form^3,6,8,11,12^. Fortificants that utilize micro- and/or nanoencapsulation can further improve bioavailability^12–14^ although the cost of using appropriately chelated and/or encapsulated Fe fortificants ($2 USD per ton to fortify wheat flour with EDTA-chelated Fe alone), and the requirement for centralized cereal processing and industrial milling limits flour fortification programs in less developed countries^8,11,15,16^. Furthermore, and perhaps more importantly, both supplementation and fortification frequently deliver excess dietary Fe to the human intestinal lumen which can cause severe gastrointestinal disruption, dysbiosis and the proliferation of non-beneficial gut bacteria^3,17–22^.

Nicotianamine (NA) is a non-protein amino acid that functions as an endogenous chelator of Fe, zinc (Zn) and other transition metals in higher plants. In graminaceous cereals NA serves as the biosynthetic precursor to 2′-deoxymugenic acid (DMA), a related Fe chelator in plant tissues that also functions as a root-secreted phytosiderophore to chelate ferric Fe in the rhizosphere^23^. Both NA and/or DMA are major Fe chelators in white wheat (*Triticum aestivum* L.) flour and enhancers of *in vitro* Fe bioavailability^24–26^ and increased NA/DMA biosynthesis has been employed to biofortify wheat^25,27^ and rice (*Oryza sativa* L.)^28–30^ with Fe and Zn. While both NA and DMA chelate ferric (Fe^3+^) ions, only NA is capable of chelating highly-bioavailable Fe^2+^ ions^31,32^. Iron biofortified rice with increased NA biosynthesis has also reversed anemia symptoms in mice, suggesting that NA-chelated Fe is bioavailable *in vivo*^33,34^. Taken together these results reveal NA-chelated Fe as a natural and highly bioavailable Fe fortificant that improves host Fe status.

The chicken (*Gallus gallus*) model is physiologically relevant for estimating dietary micronutrient absorption in humans due to similarities in intestinal morphology and enteric microbiota, and has been used in numerous studies to evaluate Fe and Zn bioavailability in staple foods^35–44^. Here we utilized the chicken model to investigate the impact of NA-chelated and EDTA-chelated Fe on Fe status and gastrointestinal health when delivered alongside extracts of control and biofortified white wheat flour containing increased NA, Fe, Zn and DMA through intraamniotic administration four days prior to hatch (short-term exposure). Short-term exposure to NA-chelated Fe and extracts of biofortified wheat flour had similar effects on gastrointestinal health; we therefore conducted a separate feeding trial study of control and biofortified wheat-based diets over a period of six weeks (long-term exposure). Together this study highlights the versatility of the chicken model and demonstrates novel positive effects of NA-chelated Fe on host Fe status and gastrointestinal health when administered as an Fe fortificant or as part of a biofortified diet.

## Results

### Experiment 1 – Intraamniotic administration of EDTA-chelated and NA-chelated Fe fortificants

#### Intraamniotic administration of NA-chelated Fe improves Fe status and alters expression of Fe homeostasis/hypertension genes

**Figure 1:**
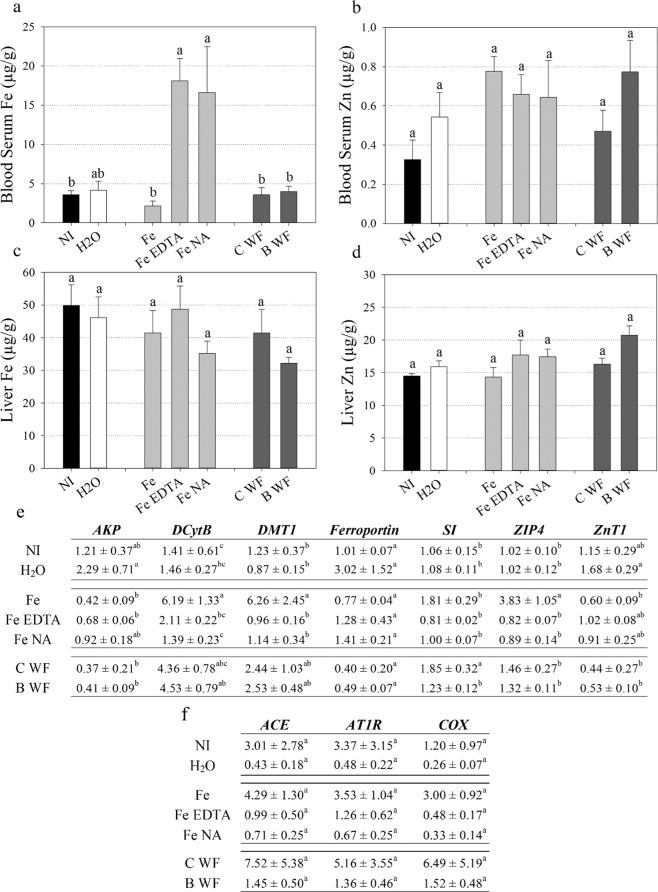
Biomarkers of Fe and Zn status following intraamniotic administration. Fe and Zn concentration (µg/g) in chicken (**a,b**) blood serum, respectively; and (**c,d**) liver, respectively. Bars represent mean ± SEM of at least three biological replicates. (**e,f**) Transcript quantification of genes in chicken duodenal and heart tissue, respectively. Values (expression ratio relative to 18S) represent mean ± SEM of at least three biological replicates, each with two technical replicates of quantitative RT-PCR. Different letters indicate significantly different values between treatment groups as analyzed by one-way ANOVA with Tukey post-hoc test (p < 0.05). NI: non-injected, C WF: control white flour extract, B WF: biofortified white flour extract.

Blood serum Fe concentration was significantly elevated in chickens that received intraamniotic administration of EDTA-chelated Fe (‘Fe EDTA’) and NA-chelated Fe (‘Fe NA’) relative to unchelated Fe (‘Fe’) and non-injected (‘NI’) treatment groups (Fig. 1A). Blood serum Zn, liver Fe and liver Zn concentrations were not significantly different between treatment groups (Fig. 1B–D).

*Duodenal cytochrome B* (*DcytB*), *divalent metal transporter 1* (*DMT1*) and *Zn transporter* (*ZIP4*) expression was significantly upregulated in intestinal tissue of chickens that received ‘Fe’ relative to all treatment groups, except for *DcytB* and *DMT1* expression in control white flour extract (‘C WF’) and biofortified white flour extract (‘B WF’) treatment groups (Fig. 1E). Both *alkaline phosphatase (AKP)* and *Zn transporter 1* (*ZnT1*) expression were significantly upregulated in chickens that received intraamniotic administration of H_2_O (‘H_2_O’) relative to ‘Fe’, ‘C WF’ and ‘B WF’ treatment groups (Fig. 1E). No differences in heart gene expression were observed between treatment groups (Fig. 1F).

#### Intraamniotic administration of NA-chelated Fe positively affects intestinal morphology and microbial population density

**Figure 2:**
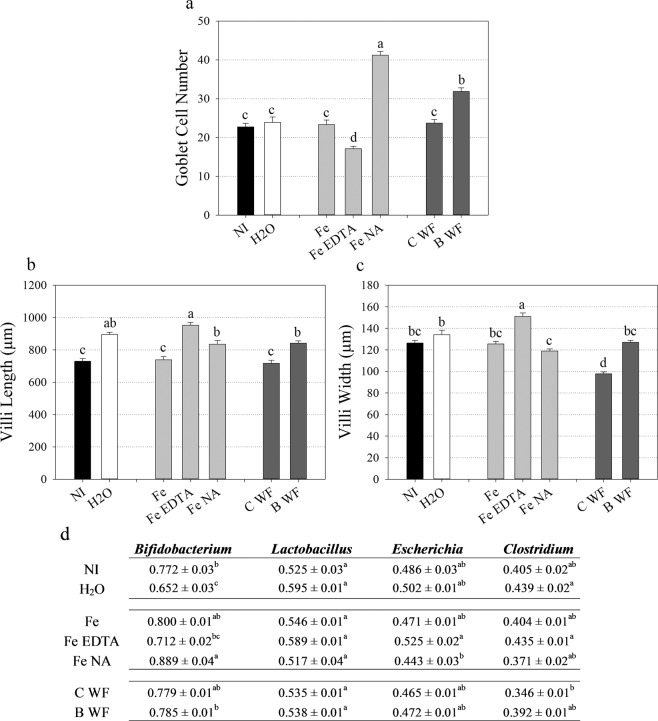
Intestinal functionality and cecal microbial composition following intraamniotic administration. (**a**) Chicken intestinal goblet cell number. (**b,c**) Chicken intestinal villi length and width (µm), respectively. Bars represent mean ± SEM of five biological replicates. (**d**) Bacterial proportions relative to a universal bacterial population present in ceca. Values (arbitrary units, AU) represent mean ± SEM of five biological replicates. Different letters indicate significantly different values between treatment groups as analyzed by one-way ANOVA with Tukey post-hoc test (p < 0.05). NI: non-injected, C WF: control white flour extract, B WF: biofortified white flour extract.

Goblet cell number increased significantly in ‘Fe NA’ intestinal villi relative to all treatment groups and in ‘B WF’ relative to all groups except for ‘Fe NA’ (Fig. 2A). Goblet cell number decreased significantly in ‘Fe EDTA’ intestinal villi relative to all treatment groups. Intestinal villi length increased significantly in ‘Fe EDTA’ relative to all treatment groups except for ‘H_2_O’ and in ‘H_2_O’, ‘Fe NA’ and ‘B WF’ treatment groups relative to ‘NI’, ‘Fe’ and ‘B WF’ treatment groups (Fig. 2B). Intestinal villi width increased significantly in ‘Fe EDTA’ relative to all treatment groups, and in ‘H_2_O’ relative to ‘Fe NA’ (Fig. 2C). Intestinal villi width decreased significantly in ‘C WF’ relative to all treatment groups.

The abundance of *Bifidobacterium* significantly increased in ‘Fe NA’ cecum relative to all treatment groups apart from ‘Fe’ and ‘C WF’, and significantly decreased in ‘H_2_O’ relative to all treatment groups apart from ‘Fe EDTA’ (Fig. 2D). The abundance of both *Escherichia* significantly increased in ‘Fe EDTA’ cecum relative to ‘Fe NA’ and *Clostridium* significantly increased in ‘H2O’ and ‘Fe EDTA’ relative to ‘C WF’ (Fig. 2D).

### Experiment 2 – Feeding trial of control and biofortified white wheat flour

#### Biofortified white wheat flour increases total body hemoglobin and hemoglobin maintenance efficiency with lower feed intake and feed conversion ratio

The concentrations of Fe, Zn, NA and DMA were significantly higher in white flour derived from field-grown bread wheat expressing the rice nicotianamine synthase (*OsNAS2*) gene compared to control white flour (Fig. S1, Table S1) and significantly increased 1.1- to 1.2-fold (Fe and Zn) and 1.7- to 1.8-fold (NA and DMA) in diet containing 80% biofortified white flour (‘Biofortified’) relative to diet containing 80% control white flour (‘Control’) (Table 1). Caco-2 cell ferritin significantly increased after exposure to biofortified white flour relative to control white flour (Fig. S2). At week 2, hemoglobin (Hb), total body Hb and hemoglobin maintenance efficiency (HME) decreased significantly in ‘Biofortified’ relative to ‘Control’ chickens (Table 2). From week 4 onwards, a trend of lower cumulative feed intake (g) and cumulative feed conversion ratio (FCR) was present in ‘Biofortified’ relative to ‘Control’ chickens. No differences in body weight between ‘Biofortified’ and ‘Control’ chickens were observed throughout the study (Table 2).

**Table 1:** Composition of the experimental diets.

Diet Ingredient	Control	Biofortified
	g/Kg (by formulation)	
Control white wheat flour	800	—
Biofortified white wheat flour	—	800
skim milk, dry	99.75	99.75
DL-methionine	2.5	2.5
corn oil	27	27
choline chloride	0.75	0.75
vitamin/mineral premix (no Fe/Zn)	70	70
**Selected Components**	**Control**	**Biofortified**
Dietary Fe (µg/g)	25.9 ± 0.12	28.9 ± 0.13***
Dietary Zn (µg/g)	16.6 ± 0.06	19.2 ± 0.03***
Dietary NA (µmol/g)	18.1 ± 0.32	33.0 ± 0.21***
Dietary DMA (µmol/g)	19.5 ± 0.16	34.1 ± 0.74***
Dietary Phytate (mg/g)	0.5 ± 0.09	0.5 ± 0.08
Total Fiber (µg/g)	19.9 ± 0.18	23.8 ± 1.12
Total Protein (%)	13.47 ± 0.08	13.67 ± 0.08
Total Carbon (%)	41.90 ± 0.13	41.30 ± 0.13
Phytate: Fe molar ratio	1.63	1.46

Component values represent mean ± SEM of at least four technical replicates. Asterisks denote significant differences for p ≤ 0.001 (***) as determined by Student’s t-test.

**Table 2:** Body weight, biomarkers of Fe status and feed consumption throughout the study.

Variable	Diet	Baseline	Week 1	Week 2	Week 3	Week 4	Week 5	Week 6
Body Weight (kg)	Control	0.125 ± 0.007	0.158 ± 0.004	0.195 ± 0.007	0.236 ± 0.010	0.286 ± 0.013	0.355 ± 0.016	0.365 ± 0.029
	Biofortified	0.122 ± 0.006	0.160 ± 0.004	0.191 ± 0.007	0.222 ± 0.010	0.260 ± 0.014	0.318 ± 0.018	0.353 ± 0.029
Hb (g/L)	Control	72.7 ± 2.3	96.5 ± 1.6	112.2 ± 1.2***	103.5 ± 3.5	99.6 ± 3.7	82.4 ± 3.7	94.9 ± 3.6
	Biofortified	72.7 ± 2.3	93.2 ± 1.6	92.5 ± 1.5	97.8 ± 3.7	104.7 ± 4.0	91.4 ± 3.4	101.2 ± 3.5
Total Body Hb (mg)	Control	2.59 ± 0.14	4.26 ± 0.124	6.15 ± 0.17***	7.09 ± 0.35	7.87 ± 0.33	8.48 ± 0.67	9.83 ± 1.01
	Biofortified	2.52 ± 0.13	4.20 ± 0.124	4.72 ± 0.17	6.21 ± 0.39	7.57 ± 0.36	7.74 ± 0.70	10.06 ± 0.98
HME (%)	Control		12.16 ± 0.879	13.80 ± 0.67***	10.21 ± 0.77	8.32 ± 0.50	4.36 ± 0.76	3.14 ± 1.02
	Biofortified		11.17 ± 0.879	7.14 ± 0.71	8.56 ± 0.85	8.41 ± 0.55	4.65 ± 0.76	5.84 ± 1.00
FCR	Control		5.85 ± 0.738	4.82 ± 0.55	6.10 ± 0.52	7.98 ± 0.51	4.09 ± 0.24	22.30 ± 3.19
	Biofortified		4.86 ± 0.736	5.98 ± 0.55	6.14 ± 0.57	6.89 ± 0.53	3.63 ± 0.25	19.81 ± 2.99
Feed Intake (g)	Control		180.6 ± 20.0	157.8 ± 13.3	251.0 ± 27.4	299.9 ± 41.9	284.5 ± 29.8	243.7 ± 25.2
	Biofortified		171.0 ± 20.0	152.6 ± 13.3	201.3 ± 27.4	244.8 ± 41.9	190.8 ± 29.8	185.6 ± 21.8
Cumulative Feed Intake (g)	Control			338.5 ± 31.6	589.5 ± 50.7	889.4 ± 91.2	1174.0 ± 119.0	1333.0 ± 153.0
	Biofortified			323.6 ± 31.6	524.9 ± 50.7	769.7 ± 91.2	960.0 ± 119.0	1096.0 ± 153.0
Cumulative FCR	Control			4.56 ± 0.38	5.00 ± 0.39	5.45 ± 0.63	4.92 ± 0.52	6.12 ± 0.85
	Biofortified			5.15 ± 0.38	5.25 ± 0.39	4.73 ± 0.63	4.16 ± 0.52	4.66 ± 0.73

Values represent mean ± SEM of at least nine biological replicates. Asterisks denote significant differences between diet treatments for p ≤ 0.001 (***) as determined by Student’s t-test. Hb: hemoglobin, HME: hemoglobin maintenance efficiency, FCR: feed conversion ratio.

#### Biofortified white wheat flour improves iron status and glycogen storage

**Figure 3:**
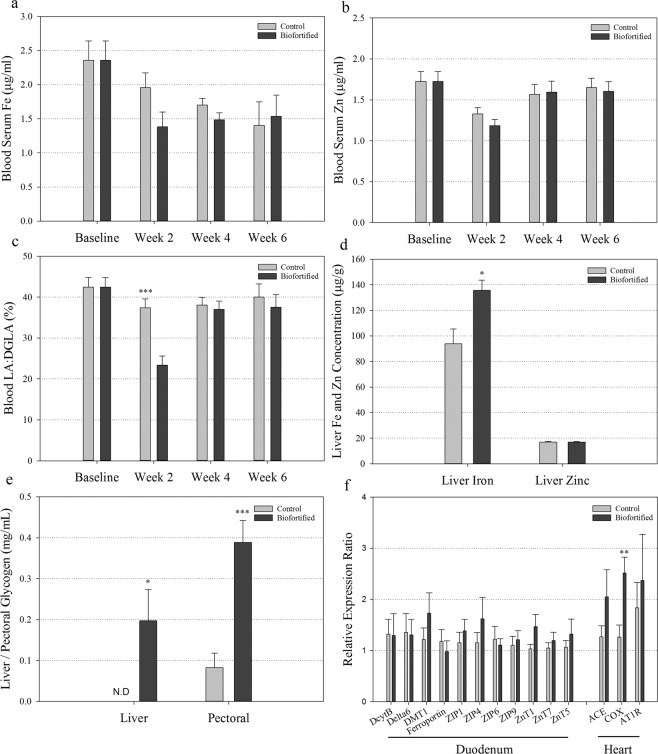
Biomarkers of Fe and Zn status and glycogen storage following consumption of experimental diets. (**a,b**) Fe and Zn concentration (µg/g) in chicken blood serum, respectively. (**c**) Ratio of LA:DGLA in chicken blood cells. Measurements were taken at the beginning (Baseline) and in the 2^nd^, 4^th^ and 6^th^ week of the study. (**d**) Fe and Zn concentration (µg/g) in chicken liver. (**e**) Glycogen (mg/mL) concentration in chicken liver and pectoral tissue. Bars represent mean ± SEM of nine biological replicates. (**f**) Transcript quantification relative to 18 S in chicken duodenal and heart tissue. Bars represent mean ± SEM of at least eight biological replicates, each with two technical replicates of quantitative RT-PCR. Asterisks denote significant differences for ^*^p < 0.05, ^***^p ≤ 0.001 as determined by Student’s t-test.

No differences in blood serum Fe and Zn concentrations were observed between ‘Biofortified’ and ‘Control’ chickens throughout the study (Fig. 3A,B). At week 2, blood linoleic acid:dihomo-γ-linolenic acid ratio (LA:DGLA) was significantly decreased in ‘Biofortified’ relative to ‘Control’ chickens (Fig. 3C). At the conclusion of the study, liver Fe concentration and glycogen storage in both liver and pectoral tissue was significantly elevated in ‘Biofortified’ relative to ‘Control’ chickens (Fig. 3D,E). No differences in nail or feather Fe and Zn concentrations were observed throughout the study (Fig. S3). Expression of *cytochrome c oxidase* (*COX*) was significantly upregulated in ‘Biofortified’ heart tissue relative to ‘Control’ (Fig. 3F).

#### Biofortified white wheat flour increases goblet cell number and positively alters gut health and the microbiome

**Figure 4:**
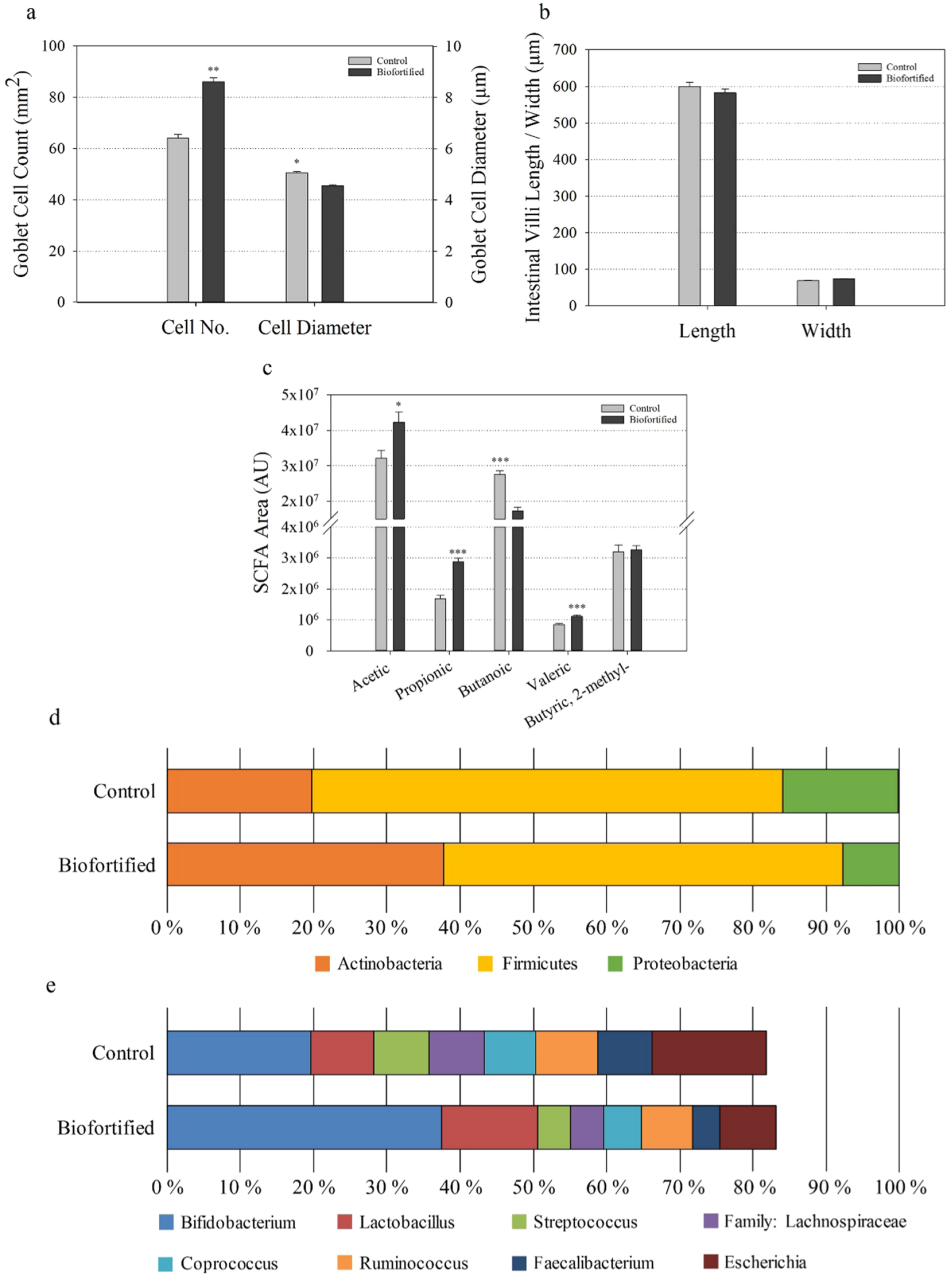
Intestinal functionality, short-chain fatty acid production and cecal microbial composition following consumption of experimental diets. (**a**) Chicken intestinal goblet cell number and diameter (µm). (**b**) Chicken intestinal villi length and width (µm). (**c**) Cecal short-chain fatty acid (SCFA) composition. Bars represent mean ± SEM of nine biological replicates. Relative abundance of microbial populations at the levels of (**d**) phyla; and (**e**) families and genera. Asterisks denote significant differences for ^*^p < 0.05, ^***^p ≤ 0.001 as determined by Student’s t-test. AU: arbitrary units.

The number of intestinal goblet cells significantly increased, the number of acidic/neutral goblet cells significantly increased, and the diameter of intestinal goblet cells significantly decreased in ‘Biofortified’ relative to ‘Control’ chickens (Figs. 4A, S4). No difference in intestinal villi length and width was detected (Fig. 4B). Short-chain fatty acid (SCFA) production significantly increased for acetic acid, propionic acid and valeric acid and decreased for butanoic acid in ‘Biofortified’ relative to ‘Control’ chickens (Fig. 4C). For major bacteria phyla the proportion of Actinobacteria increased 1.9-fold while the proportion of Firmicutes and Proteobacteria decreased 1.2- and 2.0-fold, respectively in ‘Biofortified’ ceca relative to ‘Control’ (Fig. 4D). For major bacterial genera the proportion of *Bifidobacterium* and *Lactobacillus* increased 1.9- and 1.5-fold, respectively while the proportion of *Streptococcus* (1.7-fold), *Coprococcus* (1.4-fold), *Ruminococcus* (1.2-fold) *Faecalibacterium* (2-fold), and *Escherichia* (2-fold) decreased in ‘Biofortified’ relative to ‘Control’ (Fig. 4D). The proportion of family Lachnospiraceae decreased 1.7-fold and was significantly (p = 0.045) lower in ‘Biofortified’ relative to ‘Control’ (Fig. 4D). Only one genus, *Enterococcus*, was significantly (p = 0.010) more abundant in ‘Biofortified’ (3.5%) relative to ‘Control’ (>1.0%). The abundance of all families and genera detected decreased 1.5-fold in ‘Biofortified’ cecum relative to ‘Control’.

#### Biofortified white wheat flour significantly alters diversity and metagenomic potential of the intestinal microbiota

**Figure 5:**
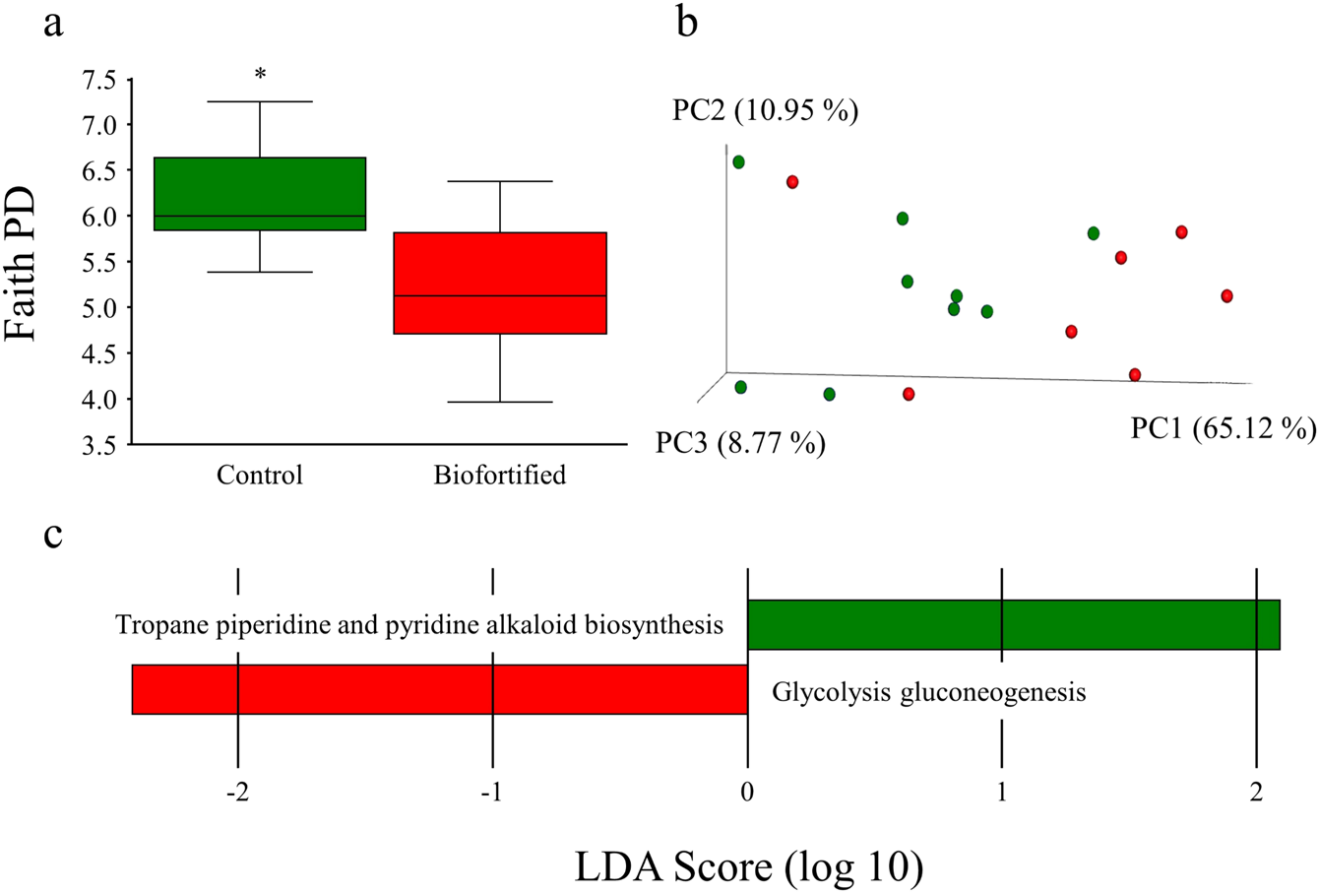
Microbial diversity and metabolic capacity following consumption of experimental diets. (**a**) Microbial α-diversity of chicken ceca using Faith’s phylogenetic diversity (PD). (**b**) Microbial β-diversity of chicken ceca using unweighted UniFrac distances separated by three principal components (PC). Each dot represents either a ‘Control’ (green) or ‘Biofortified’ (red) chicken. (**c-d**) computed linear discriminant analysis (LDA) scores of differences in microbial relative abundance and metabolic capacity, respectively. Positive LDA scores (green) are enriched in ‘Control’ and negative LDA scores (red) are enriched in ‘Biofortified’. Asterisks denote significant differences for ^*^p < 0.05 as determined by Kruskal-Wallis test.

Microbial population diversity (α-diversity) represented as Faith’s phylogenetic diversity significantly decreased in ‘Biofortified’ cecum relative to ‘Control’ (Fig. 5A). Significant (q = 0.042) separate clustering (β-diversity) of weighted ‘Biofortified’ and ‘Control’ microbial populations was observed (Fig. 5B) with family Enterococcaceae (including an unspecified genus) significantly more abundant and genus *Dorea* significantly less abundant in ‘Biofortified’ relative to ‘Control’ (Fig. S5). Microbial glycolysis/gluconeogenesis significantly increased and microbial tropane piperidine and pyridine alkaloid biosynthesis significantly decreased in ‘Biofortified’ microbial populations relative to ‘Control’ (Fig. 5C).

## Discussion

Both NA and DMA form high affinity 1:1 complexes with Fe^3+^ (formation constants of 10^20^ and 10^18^, respectively) and NA complexes Fe^2+^ with a formation constant of 10^13^ ^31,32^. By contrast, EDTA forms a pentagonal bipyramidal complex surrounding a single Fe^3+^ atom with a formation constant of 10^25^, and likely provides Fe^3+^ ions to the small intestine that require reduction by *DCytB* before absorption^31,45,46^. Intraamniotic administration of ‘Fe EDTA’ and ‘Fe NA’ significantly increased blood serum Fe and significantly downregulated the expression of intestinal *DMT1* (a major Fe transporter) relative to administration of unchelated ‘Fe’ (Fig. 1A,E) suggesting that both EDTA-chelated and NA-chelated Fe are readily absorbed into the small intestine before export into the blood stream^3,47^. Decreased expression of *DCytB* (which catalyzes the reduction of Fe^3+^ to Fe^2+^) in ‘Fe NA’ relative to unchelated ‘Fe’ is evidence that NA delivers relatively more Fe^2+^ ions, and that administration of an unchelated Fe solution delivers relatively more oxidized Fe^3+^ ions to the intestine^48^. The expression of *Ferroportin* (the only known intestinal Fe exporter) was similar between treatment groups, and determining whether these NA-chelated Fe^2+^ ions would be preferentially absorbed into intestinal enterocytes or transferred paracellularly into the blood stream requires further investigation^47^. Given that low expression of duodenal *DMT1*/*DCytB* relative to *Ferroportin* is linked with a positive gut microbiome^49^ and that ‘Fe NA’ administration resulted in proliferation of probiotic *Bifidobacterium* in the ceca relative to *Escherichia* and *Clostridium* (Fig. 2D), we hypothesize that NA-chelated Fe is readily absorbed by the host and not available to Fe-responsive pathogenic bacteria^20,21,50–52^. Increased cecal *Escherichia* abundance in ‘Fe EDTA’ instead suggests that EDTA-chelated Fe persists in the intestinal lumen and contributes to the proliferation of non-beneficial bacteria (Fig. 2D). Within the intestine, goblet cells are responsible for the synthesis and secretion of mucus, a polysaccharide/protein rich layer that physically protects epithelial cells, provides microbial habitat and facilitates nutrient exchange^53–55^. We hypothesize that highly bioavailable NA-chelated Fe is readily absorbed by the intestinal epithelia, leading to significantly increased goblet cell number (Fig. 2A) and a mucosal habitat that supports probiotic *Bifidobacterium*^56^. By contrast, reduced intestinal goblet cells (and mucus production) coupled with increased villi surface area in ‘Fe EDTA’ may amplify the risk of bacterial infection due an increased proportion of potentially pathogenic *Escherichia* and *Clostridium* relative to probiotic *Bifidobacterium*^55^. Together these results suggest NA-chelated Fe is highly bioavailable to the host and improves intestinal functionality without causing dysbiosis and proliferation of pathogenic bacteria as commonly seen in traditional Fe supplements and fortificants^20,22^.

Biofortification is a cost-effective strategy to combat human micronutrient deficiencies by improving the density and/or bioavailability of micronutrients in staple crops through agronomic practices, conventional breeding, or modern biotechnology^57,58^. Biofortification efforts in pearl millet (*Pennisetum glaucum* L.) and common bean (*Phaseolus vulgaris* L.) have increased seed Fe concentration (up to 3.9-fold and 1.7-fold, respectively), and consuming these crops improves both Fe status and cognitive performance^59–62^. Traditional biomarkers of Fe status including blood serum Fe concentration and Fe homeostasis gene expression^47^ were unchanged between chickens that received ‘Biofortified’ and ‘Control’ diets (Fig. 3A,F). Caco-2 cell ferritin formation, a commonly used biomarker for measuring Fe bioavailability *in vitro*^37^, increased in biofortified white flour relative to control white flour but did not differ between digests of ‘Biofortified’ and ‘Control’ diets (Fig. S2). Instead we observed a trend of increasing blood Hb and HME from week 4 onwards (Table 2), and significantly increased liver Fe concentration and heart *COX* gene expression^63^ at week 6 (Fig. 3D,F), indicating that ‘Biofortified’ chickens had improved Fe status relative to ‘Control’ chickens and demonstrating the importance of a holistic approach in evaluating host Fe status^37^. Interestingly we observed significantly decreased blood Hb, total body Hb and HME in ‘Biofortified’ chickens relative to ‘Control’ chickens at week 2 (Table 2). The ‘Control’ chicken blood Hb and HME values at week 2 were the highest values obtained throughout the study and may be evidence of a carryover effect from consuming a nutrient-rich commercial diet prior to commencement of the study. We hypothesize that ‘Control’ chickens utilized greater amounts of Fe (and likely other nutrients) from the commercial diet as part of an adaptive response to the poor nutrient concentrations within the ‘Control’ diet (Table 1), and that ‘Biofortified’ chickens did not utilize the additional Fe within this commercial diet to the same extent. Together these results highlight the importance of conducting long-term feeding studies to accurately evaluate biofortified diets and more comprehensive investigation of this hypothetical nutrient utilization mechanism is warranted. Short-term exposure to extracts of biofortified white flour was insufficient to alter liver Fe storage in ‘B WF’ (Fig. 1C) and further highlights the importance of long-term exposure when evaluating biofortified diets. Given the large difference in feed consumption (Table 2) and relatively small (3 ppm) difference in dietary Fe concentration, ‘Biofortified’ chickens had lower Fe intake than ‘Control’ chickens over the course of the study (31.6 mg compared to 34.5 mg Fe). The increased liver Fe concentration to ‘Biofortified’ chickens relative to ‘Control’ chickens must therefore be the result of improved Fe bioavailability in the biofortified diet, likely due to increased concentration of NA- and/or DMA-chelated Fe given that both NA and DMA enhance Fe bioavailability *in vitro*^26,33,34^. Separating the effect of NA and DMA on dietary Fe bioavailability requires a follow-up study evaluating diets fortified with NA-chelated or DMA-chelated Fe and together these results reinforce the importance of the chelated form of Fe rather than target levels as a consideration for future Fe fortification and biofortification programs.

Here we show for the first time that the benefits of consuming a biofortified diet include altered intestinal functionality, enteric microbiota and feed energy conversion. Biofortified wheat consumption increased the abundance of *Bifidobacterium* and *Lactobacillus* in ‘Biofortified’ ceca relative to Clostridales (comprising *Coprococcus Ruminococcus, Faecalibacterium* and family Lachnospiraceae) and *Escherchia* (Fig. 4E) which is strikingly similar to the results obtaining following intraamniotic administration of NA-chelated Fe (Fig. 2D) and provides further evidence that NA- and/or DMA-chelated Fe is highly bioavailable and does not persist in the intestinal lumen where it can contribute to the proliferation of pathogenic bacteria^51,52^. The major phyla observed in this study: Firmicutes, Actinobacteria and Proteobacteria are shared between humans and chickens^36,64^. Typically Firmicutes are the most abundant (70–80%) and Actinobacteria least abundant (~5%) phyla in human and poultry, suggesting the atypical microbial composition of both ‘Control’ and ‘Biofortified’ (~20% and 38% Actinobacteria, respectively) is due to nutritional insufficiencies in both diets^65–67^. *Bifidobacterium* and *Lactobacillus* are major probiotic genera within Actinobacteria and Firmicutes respectively, and both genera symbiotically harvest additional nutrients and energy from the diet for the host^68,69^. These probiotic populations likely inhabit the additional intestinal mucin secreted by increased goblet cells in ‘Biofortified’ chickens (Figs. 4A, S4) that are both acidic and neutral and provide mucin with an appropriate chemical composition to support these populations^70^. We hypothesize that additional *Bifidobacterium* and *Lactobacilli* in the mucosal layer upregulate glycolysis/gluconeogenesis enzymes and increase the production of acetic, propionic and valeric SCFAs (Figs. 4C, 5C), leading to improved host Fe absorption and carbohydrate metabolism in ‘Biofortified’ chickens relative to ‘Control’^66,69,71^. Improved metabolic capacity in ‘Biofortified’ chickens manifested as reduced cumulative FCR (consuming ~20% less for the same weight gain) and increased glycogen storage in both liver and pectoral tissues relative to ‘Control’ (Table 2, Fig. 3E). Improved food energy conversion due to increased *Bifidobacterium*/*Lactobacillus* relative to *Escherchia* was observed following prebiotic supplementation in broiler chickens^72^, suggesting these effects may be due to NA and/or DMA acting as prebiotics in the biofortified diet (Table 1). Administering extracts of biofortified white flour (containing NA and DMA) increased intestinal goblet cell number and villi surface in ‘B WF’ relative to ‘C WF’ (Figs. 1D,E, 2), suggesting that even short-term exposure to biofortified wheat positively affects intestinal morphology.

Traditional biomarkers of Zn status such as *ZIP4* and *ZnT1* gene expression and Zn concentration in blood serum, nails, and feathers^39,73^ were unchanged in ‘Biofortified’ chickens relative to ‘Control’, suggesting that Zn status was also unchanged (Figs. 3B,F, S3). Given the small differences in dietary Zn concentration (<3 ppm), ‘Biofortified’ chickens had lower Zn consumption than ‘Control’ chickens over the course of the study (21.0 mg compared to 22.1 mg Zn, respectively). Together these results suggest that ‘Biofortified’ chickens had improved Zn bioavailability likely due to consumption of increased dietary NA and/or DMA, although whether NA and/or DMA increase Zn bioavailability requires further investigation. We observed significantly decreased LA:DGLA at week 2 and a trend of decreased LA:DGLA from week 4 onwards in ‘Biofortified’ relative to ‘Control’ (Fig. 3C). As the LA:DGLA is a sensitive novel biomarker for evaluating Zn status^74^, these results suggest that longer-term (6 months) exposure to ‘Biofortified’ diet may demonstrate clearer improvements to Zn status and is warranted. Zinc deficiency in chickens is known to negatively alter the gut microbiome, and improved microbial composition in ‘Biofortified’ chickens may be an additional symptom of improved Zn status^75^. Given NA and DMA enhance Fe bioavailability we have previously argued these natural metal chelators function as phytonutrients in cereal foods^25,26,76^. It is well established that NA exhibits anti-hypertensive effects *in vivo*^34,77^ although we did not detect differences in heart *angiotensin-converting enzyme* (*ACE*) and *angiotensin II receptor type 1* (*AT1R*) gene expression throughout our study (Figs. 1F, 3F). We suspect similar heart *ACE* and *AT1R* expression between treatment groups in both the intraamniotic administration and feeding trial experiments is due to the relatively short exposure time to Fe solutions (4 days) or experimental diets (6 weeks) and it is worth investigating whether longer-term (6 months) exposure to increased dietary NA reduces hypertension. Nevertheless, the improved Fe status, gastrointestinal health and microbial composition in chickens following short and long-term exposure to NA-chelated Fe reinforces the idea of NA as an important phytonutrient in plant foods. Furthermore, utilization of NA-chelated Fe in food fortification and crop biofortification programs shows great potential to improve human health.

## Materials and Methods

### Plant material and white flour production

Vector construction, plant transformation and the initial selection of biofortified wheat material is described in^25^. In brief, the full-length coding sequence of *OsNAS2* (LOC_Os03g19420) was PCR amplified from rice (*Orzya sativa* L.) cv. Nipponbare and recombined into a modified pMDC32 vector under transcriptional control of the maize (*Zea mays* L.) ubiquitin 1 (UBI-1) promoter with a hygromycin phosphotransferase plant-selectable marker (Fig. S1). Bombardment of the construct into immature wheat (*Triticum aestivum* L.) cv. Bobwhite embryos was performed at the University of Adelaide (Adelaide, Australia). One double-insert event and corresponding null segregant (termed ‘Biofortified’ and ‘Control’, respectively) were grown were grown for two seasons for use in intra-amniotic administration (2016 field season) and feeding trial (2017 field season) in New Genes for New Environment facilities located in Merredin, Western Australia (Fig. S1, Table S1). Whole grain samples from Merredin were conditioned to 15% moisture content and milled (70–75% extraction) using a Quadrumat Junior laboratory mill (Brabender, Duisburg, Germany) for intraamniotic administration or a Buhler MLU-202 laboratory mill at The Commonwealth Scientific and Industrial Research Organisation (CSIRO, ACT, Australia) for the feeding trial. All break and reduction fractions of ‘Biofortified’ or ‘Control’ grain were combined to form either ‘Biofortified’ white flour or ‘Control’ white flour (Table 1).

### Preparation of extracts, solutions and diets

Wheat extracts were generated as described in^78^. In brief, ‘Biofortified’ white flour or ‘Control’ white flour was mixed in dH_2_O (50 g/L), filtered (600 µm) and centrifuged, and the resulting supernatant was dialyzed (MWCO 12–14 kDa, Medicell International Ltd., London, UK) exhaustively against dH_2_O (48 hrs.). The dialysate was lyophilized, and the resulting powder dissolved in 18MΩ H_2_O (0.05 g/mL) forming the white wheat flour extracts for intra-amniotic administration. Iron solutions were prepared by combining an Fe standard (1000 μg/mL, 2% HCl, High-Purity Standards, Charleston, SC, USA) with either 18MΩ H_2_O (‘Fe’), or 1.6 mM NA (Toronto Research Chemicals Inc., Toronto, Canada) dissolved in 18MΩ H_2_O (‘Fe NA’). The (‘Fe EDTA’) solution was achieved by combining ferric nitrate (Fe(NO_3_)_3_ 9H_2_O, Sigma, St. Louis, MO, USA) with hydroxyethyl ethylenediamine triacetic acid (H_3_HEDTA, Sigma, St. Louis, MO, USA) dissolved in sodium hydroxide (NaOH, Sigma, St. Louis, MO, USA) to represent an anionic chelate of dissolved NaFeEDTA^45^ with final Fe concentration of 77 µM. Osmolarity and final Fe concentration of extracts/Fe solutions for intraamniotic administration is provided (Table S2).

### Dietary analysis (phytate, protein, carbon, fiber, NA, DMA)

Dietary phytate was calculated relative to total phosphorus released from diet and flour samples by phytase and alkaline phosphatase enzymes according to manufacturer’s instructions (K-PHYT 11/15. Megazyme International. Bray, Ireland). Total dietary carbon (%) and nitrogen (%) was measured via Dumas combustion using a TruMac® CN (LECO Corporation, St. Joseph, MI, USA) with total protein (%) for wheat diet samples equal to 5.7 × total nitrogen (%). Total dietary fiber was measured via enzymatic digestion using heat-resistant amylase, protease and amyloglucosidase according to manufacturer’s instructions (Total Dietary Fiber Assay Kit, Sigma, St. Louis, MO, USA). Quantification of NA and DMA in diet and flour samples was performed as described in^25^. Briefly, sequential MeOH (100%) and 18MΩ H_2_O sample were derivatized by 9-fluorenylmethoxycarboxyl chloride (FMOC-Cl) and quantified via RP LC-MS on a 1290 Infinity II and 6490 Triple Quadrupole LC/MS system (Agilent Technologies Inc., Santa Clara, CA, USA).

### Caco-2 Fe bioavailability bioassay

Diet and flour samples were subjected to the Caco-2 cell bioassay as previously described^25^. Briefly, gastric-digested samples (1.5 mL) were added to cylindrical Transwell inserts (Corning Life Sciences, Corning, NY) fitted with a semipermeable (15 000 Da MWCO) basal membrane (Spectra/Por 2.1, Spectrum Medical, Gardena, CA) and placed within wells containing Caco-2 cell monolayers. Following overnight incubation, cells were washed, harvested and analyzed for ferritin (FER-IRON II Ferritin Assay, Ramco Laboratories, Houston, TX) and total protein contents (Bio-Rad DC Protein Assay, Bio-Rad, Hercules, CA). As Caco-2 cells synthesize ferritin in response to intracellular Fe, we used the ratio of ferritin/total protein (expressed as ng ferritin/mg protein) as an index of cellular Fe uptake.

### Micronutrient analysis

Micronutrient concentration in white flour, diets and extracts, Fe solutions, blood serum, and all animal tissues was determined by nitric/perchloric acid digestion as previously described^38^ followed by inductively coupled plasma-optical emission spectrometry (ICP-OES) using a Thermo iCAP 6500 series (Thermo Jarrell Ash Corp., Franklin, MA, USA).

### Animals and study design

Cornish-cross fertile broiler eggs (n = 70) were obtained from a commercial hatchery (Moyer’s chicks, Quakertown, PA, USA) and incubated at the Cornell University Animal Science poultry farm until hatching. All animal protocols were approved by Cornell University Institutional Animal Care and Use committee (protocol number: 2007–0129). All methods were performed in accordance with the relevant guidelines and regulations. For intraamniotic administration, eggs (n = 40) containing viable embryos were weighed and randomly assigned to seven groups (n ≥ 5) based on weight distribution. At day 17 of incubation, extracts/Fe solutions (1 mL) were injected into the amniotic fluid via a 21-gauge needle for the seven treatment groups as follows: (1) non-injected (NI); (2) 18MΩ H_2_O (H_2_O); (3) Fe solution (Fe); (4) Fe-EDTA solution (Fe-EDTA); (5) Fe-NA solution (Fe-NA); (6) ‘Control’ white flour extract (C WF); (7) ‘Biofortified’ white flour extract (B WF) and eggs were subsequently incubated for four days until hatch as described in^79,80^. Chicks were euthanized by CO_2_ exposure after hatching and all tissues were collected. The remaining hatchlings (n = 30) were allocated based on body weight into two treatment groups: (1) 80% ‘Control’ white flour diet (‘Control’) and (2) 80% ‘Biofortified’ white flour diet (‘Biofortified’) as described in^38^. All chickens received a commercial diet (Nutrena® Chick Starter Grower 18% Crumble, Cargill Inc, Wayzata, MN, USA) for one week prior to consumption of ‘Control’ and ‘Biofortified’ diets for six weeks. ‘Control and Biofortified’ diet formulations met the Nutrient Requirements for Poultry (NRC Poultry reference) excluding Fe and Zn. Chickens (n = 3) were housed in cages (1 m^2^) and provided *ad libitum* access to food and H_2_O. Feed intakes were measured daily, and body weight and blood samples were obtained weekly. Feed conversion ratio (FCR) represents weekly feed intake (g) proportional to the weekly increase in body weight (g). Chickens were euthanized by CO_2_ exposure seven weeks post-hatch and tissues collected.

### Blood measurements

Wing-vein blood samples (100 µL) were collected using micro-hematocrit heparinized capillary tubes (Fisher, Pittsburgh, PA, USA). Blood plasma Hb concentrations were determined spectrophotometrically using the Triton®/NaOH method according to manufacturer’s instructions (Hemoglobin Assay Kit, Sigma, St. Louis, MO, USA). The Hb maintenance efficiency (HME) was calculated as previously described^38^. Blood serum Linoleic Acid:Dihomo-γ-Linolenic Acid ratio (LA:DGLA) was determined as previously described^39^.

### Gene expression analysis (Tissue harvesting, RNA isolation, cDNA synthesis, primer design)

Total RNA extraction from duodenal and heart tissue (30 mg) using Qiagen RNeasy Mini Kit (RNeasy Mini Kit, Qiagen Inc., Valencia, CA, USA), cDNA synthesis and real time-polymerase chain reaction (RT-PCR) analysis were performed as previously described^38,81^ with minor adjustments. In brief, the cycle product (Cp) of each gene was quantified using a seven-point standard curve in duplicate. Gene expression was obtained relative to 18 S (Cp), primer pair efficiency, and control treatments: ‘NI’ for intraamniotic administration and ‘Control’ for feeding trial^82^. *Alkaline phosphatase* (*AKP*) and *sucrase isomaltase* (*SI*) acted as intestinal reference genes following intraamniotic administration (Fig. 1E). All primers used for gene expression analysis are provided in Table S3.

### Ferritin and glycogen analysis

Liver ferritin was determined as previously described^78^. In brief, samples (1 g) were homogenized in 4-(2-hydroxyethyl)-1-piperazineethanesulfonic acid (HEPES) buffer (50 mM) and heat treated (75 °C, 10 min) before centrifugation. Native polyacrylamide gel electrophoresis (PAGE) gels were stained with Coomassie blue G-250 stain or potassium ferricyanide [K_3_Fe(CN)_6_] and quantified using the Quantity-One 1-D analysis program (Bio-Rad, Hercules, CA). Liver and pectoral glycogen was determined colorimetrically as described in^83^ with minor adjustments. After centrifugation and mixing with petroleum ether, homogenized tissue was mixed with color reagent (300 µL) and total glycogen determined on an ELISA plate reader (450 nm) according to a standard curve.

### Intestinal functionality and short-chain fatty acid (SCFA) analysis

Duodenal samples were fixed in fresh 4% (v/v) buffered formaldehyde, dehydrated, and embedded in paraffin as previously described^38^. Serial sections (5 µm) were deparaffinized in xylene and stained with hematoxylin and eosin before goblet cell number and villi surface area examination under light microscopy using EPIX XCAP software (Standard version, Olympus, Waltham, MA, USA). Cecal samples were homogenized in HCl (2 ml, 3%, 1 M), centrifuged and combined with ethyl acetate (100 µL) and acetic acid-d4 (1 µg/mL) before collecting the organic phase to determine short chain fatty acid (SCFA) composition. Samples were quantified via GC-MS using a TRACE™ 1310 gas chromatograph (Thermo Fisher Scientific, Waltham, MA, USA) and a TraceGOLD™ TG-WaxMS A column (Thermo Fisher Scientific, Waltham, MA, USA).

### Microbial population analysis

*Lactobacillus*, *Bifidobacterium*, *Escherichia*, and *Clostridium* density in intraamniotic administration treatment groups was determined as previously described^79^. In brief, cecal contents were homogenized with phosphate-buffered saline (PBS, 9 ml), centrifuged and the pellet resuspended in ethylenediaminetetraacetic acid (EDTA, 50 mM) and treated (37 °C, 45 min) with lysozyme (10 mg/mL, Sigma Aldrich CO., St. Louis, MO, USA). Bacterial genomic DNA was isolated according to manufacturer’s instructions (Wizard® Genomic DNA Purification Kit, Promega Corp., Madison, WI, USA) and bacterial genera are presented in relative proportions. All primers used for microbial population analysis are provided in Table S4.

### 16S rRNA gene sequencing and analysis

Microbial genomic DNA extraction from ‘Control’ and ‘Biofortified’ cecal samples, gene sequencing and analysis was conducted as previously described^38^. In brief, 16S rRNA gene sequences were amplified from the V4 hypervariable region of microbial genomic DNA (Powersoil DNA isolation kit, MoBio Laboratories Ltd., Carlsbad, CA, USA, purified (AMPure, Beckman Coulter, Atlanta, GA, USA) and quantified according to manufacturer’s instructions (Quant-iT™ PicoGreen™ dsDNA Assay Kit, Invitrogen, Carlsbad, CA, USA). Samples were sequenced at Bar Ilan University (Safed, Israel) using an Illumina MiSeq Sequencer (Illumina, Inc., Madison, WI, USA). Amplicon reads were analyzed using ‘Divisive Amplicon Denoising Algorithm’ (DADA2) and ‘quantitative insights into microbial ecology’ (QIIME) software before taxonomic classification using Greengenes database (http://greengenes.lbl.gov)^84–86^. Faith’s phylogenetic diversity (PD) was used to assess α-diversity and principal component (PC) analysis of weighted UniFrac distances was used to assess β-diversity^87,88^. Relative abundance was determined using linear discriminant analysis effect size (LEfSe) and metabolic capacity was determined using ‘phylogenetic investigation of communities by reconstruction of unobserved states’ (PICRUSt) software compared to known pathways in the Kyoto Encyclopedia of Genes and Genomes (KEGG) database (https://www.genome.jp/kegg)^89,90^.

### Statistical analyses

A mixed linear model was utilized to normalize body weight, Hb, total body Hb, HME, FCR and feed intake relative to baseline total body Hb as presented in Table 2 using MiniTab software (v 18.0, MiniTab). Significant differences between intraamniotic administration treatment groups was determined by ANOVA with a Tukey post-hoc test (p < 0.05) with additional significant differences between ‘Fe’, ‘Fe EDTA’ and ‘Fe NA’ as well as between ‘C WF’ and ‘B WF’ determined by Student’s t-test (p < 0.05). Significant differences in physiological measurements between ‘Control’ and ‘Biofortified’ samples were determined by Student’s t-test (p < 0.05). Significant differences in Faith’s PD and weighted UniFrac distances was determined by Kruskal-Wallis and permutational multivariate analysis of variance (PERMANOVA) tests, respectively and LEfSe significant differences were corrected for false discovery rate (FDR).

## Supplementary information

Supplementary Material.

## Data Availability

The data that support the findings of this study are available from the corresponding author upon reasonable request.
