# Supplementary material for: Nicotianamine-chelated iron positively affects iron status, intestinal morphology and microbial populations in vivo (Gallus gallus)
Source: Sci Rep. 2020 Feb 10;10:2297. doi: 10.1038/s41598-020-57598-3 (PMC7010747; doi:10.1038/s41598-020-57598-3)
Supplement: Supplementary file 1 — Supplementary Material. [file 41598_2020_57598_MOESM1_ESM.docx]

**Nicotianamine-chelated iron positively affects iron status, intestinal morphology and microbial populations *in vivo* (*Gallus gallus*)**

Jesse T. Beasley^a^, Alexander A. T. Johnson^a^, Nikolai Kolba^b^, Julien P. Bonneau^a^, Raymond P. Glahn^b^, Lital Ozeri^c^, Omry Koren^c^ and Elad Tako^b*^

^a^School of BioSciences, The University of Melbourne, Victoria 3010, Australia

^b^Robert W. Holley Center for Agriculture and Health, USDA-ARS, Ithaca, New York 14853, USA

^c^Azrieli Faculty of Medicine, Bar-Ilan University, Safed 1311502, Israel

*****Correspondence: Tel.: +1 607-255-5434, E.: [et79@cornell.edu](mailto:et79@cornell.edu); [elad.tako@ars.usda.gov](mailto:elad.tako@ars.usda.gov)

**Supplementary Material**

**Figure S1. Generation of plant material and white flour.** (**a**) Schematic representation of the T‐DNA construct used to transform wheat (*Triticum aestivum* L.) as previously described^1^. RB and LB: right and left borders, respectively; UBI‐1: *Zea Mays* *ubiquitin 1* gene promoter; *OsNAS2*: *Oryza sativa* *nicotianamine synthase 2* gene (LOC_Os03 g19420); NOS: nopaline synthase terminator; 2 x 35S: dual promoter of 35S cauliflower mosaic virus gene; hyg: *hygromycin phosphotransferase* gene (selectable marker); 35S: 35S cauliflower mosaic virus gene terminator. (**b**) Confined field trial of ‘Biofortified’ and ‘Control’ in the New Genes for New Environment facilities located in Merredin, Western Australia. (**c**) ‘Biofortified’ and ‘Control’ wheat grown at Merredin in 2016 was milled using a Quadrumat Junior laboratory mill and extracts of the resulting white flour used in the intraamniotic administration assay. The image was adapted from^2^ with permission from the authors. (**d**) ‘Control’ and ‘Biofortified’ wheat grown at Merredin in 2017 was milled using a Buhler MLU-202 laboratory mill and the resulting white flour mixed into ‘Control’ and ‘Biofortified’ (GM) diets for a six-week long feeding trial.

**Figure S2. Iron bioavailability in Control and Biofortified white flour and experimental diets.** Bars represent mean ± SEM of three biological replicates and asterisks denote the significance between Control and Biofortified flour for p < 0.05 (*) as determined by Students’ *t*-test.

**Figure S3. Nail and feather Fe and Zn concentration following consumption of experimental diets.** The concentration (µg/g) of (**a**) Fe and (**b**) Zn in Control and Biofortified chicken nail and feather is provided. Bars represent mean ± SEM of nine biological replicates.

**Figure S4.** **Chicken intestinal goblet cell type following consumption of experimental diets.** The number of acidic, neutral and both acidic and neutral (mixture) goblet cells between Control and Biofortified chicken intestinal (**a**) villi and (**b**) crypts is provided. Bars represent mean ± SEM of nine biological replicates. Asterisks denote significant differences for p < 0.05 (*), p ≤ 0.001 (***) as determined by Student’s t-test.

**Figure S5. Differentially enriched taxa following consumption of experimental diets.** Computed linear discriminant analysis (LDA) scores of differences in microbial relative abundance. Positive LDA scores (green) are enriched in ‘Control’ and negative LDA scores (red) are enriched in ‘Biofortified’.

**Table S1. Nutritional composition of Control and Biofortified white flour.** Values represent mean ± SEM of at least four technical replicates. Asterisks denote significant differences for p ≤ 0.001 (***) as determined by Student’s t-test.

| **Selected Components** | **Control** | **Biofortified** |
| --- | --- | --- |
| White Flour Fe (µg/g) | 19.0 ± 0.07 | 24.5 ± 0.14*** |
| White Flour Zn (µg/g) | 16.0 ± 0.06 | 21.1 ± 0.03*** |
| White Flour NA (µmol/g) | 19.0 ± 0.21 | 37.4 ± 0.88*** |
| White Flour DMA (µmol/g) | 17.7 ± 0.09 | 23.6 ± 0.20*** |
| White Flour Phytate (mg/g) | 2.0 ± 0.03 | 1.9 ± 0.05 |
| Phytate : Fe molar ratio | 8.91 | 6.56 |

**Table S2.** Osmolarity and Fe concentration of extracts / Fe solutions for intraamniotic administration.

| **Group** | **Osmolality** | **Fe (µg/g)** |
| --- | --- | --- |
| NI | - | - |
| H_2_O | 87 | ND |
| Fe | 168 | 11.68 |
| Fe EDTA | 82 | 11.19 |
| Fe NA | 188 | 11.68 |
| C WF | 129 | 0.91 |
| B WF | 120 | 0.82 |

**Table S3.** The sequences of the qPCR primers used for gene expression analysis. The PCR product size and GenInfo identifier for each gene is provided.

| **Gene** | **Forward primer (5'-3')** | **Reverse primer (5'-3')** | **Product size (bp)** | **GenInfo identifier** |
| --- | --- | --- | --- | --- |
| 18s rRNA | GCAAGACGAACTAAAGCGAAAG | TCGGAACTACGACGGTATCT | 100 | 7262899 |
| ACE | CATGGCCTTGTCTGTCTCC | GAGGTATCCAAAGGGCAGG | 142 | 424059 |
| AKP | CTCATTCCAGCGTACTCTTCTT | GTGTGTAGATCAAAGGGCTACT | 100 | 424936 |
| AT1R | TCATCTGGCTCCTTGCTGG | AACCTAGCCCAACCCTCAG | 138 | 396065 |
| COX | GCAGG GTTTCCTCCAT | GGTTGCGGTCGGTAAGT | 150 | 420624 |
| Delta6 | GGCGAAAGTCAGCCTATTGA | AGGTGGGAAGATGAGGAAGA | 93 | 261865208 |
| DcytB | CATGTGCATTCTCTTCCAAAGTC | CTCCTTGGTGACCGCATTAT | 103 | 20380692 |
| DMT1 | TTGATTCAGAGCCTCCCATTAG | GCGAGGAGTAGGCTTGTATTT | 101 | 206597489 |
| Ferroportin | CTCAGCAATCACTGGCATCA | ACTGGGCAACTCCAGAAATAAG | 98 | 61098365 |
| SI | CCAGCAATGCCAGCATATTG | CGGTTTCTCCTTACCACTTCTT | 95 | 2246388 |
| ZIP1 | TGCCTCAGTTTCCCTCAC | GGCTCTTAAGGGCACTTCT | 144 | 107055139 |
| ZIP4 | TCTCCTTAGCAGACAATTGAG | GTGACAAACAAGTAGGCGAAAC | 95 | 107050877 |
| ZIP6 | GCTACTGGGTAATGGTGAAGAA | GCTGTGCCAGAACTGTAGAA | 99 | 66735072 |
| ZIP9 | CTAAGCAAGAGCAGCAAAGAAG | CATGAACTGTGGCAACGTAAAG | 100 | 237874618 |
| ZnT1 | GGTAACAGAGCTGCCTTAACT | GGTAACAGAGCTGCCTTAACT | 105 | 54109718 |
| ZnT5 | TGGTTGGTATCTGTGCCTTTAG | GGCTGTGTCCATGGTAAGATT | 99 | 56555150 |
| ZnT7 | GGAAGATGTCAGGATGGTTCA | CGAAGGACAAATTGAGGCAAAG | 87 | 56555152 |

18S rRNA, 18S ribosomal subunit; ACE, angiotensin-converting enzyme; AKP, alkaline phosphatase; AT1R, angiotensin II receptor type 1; COX, cytochrome c oxidase; Delta6, delta-6-desaturase; DcytB, duodenal cytochrome b; DMT1, divalent metal transporter 1; SI, Sucrose isomaltase; ZIP1, Zinc transporter 1; ZIP4, Zinc transporter 4; ZIP6, Zinc transporter 6; ZIP9, Zinc transporter 9; ZnT 1, Zinc transporter 1; Znt 5, Zinc transporter 5; Znt 7, Zinc transporter 7.

**Table S4.** The sequences of the primers used for microbial analysis as previously described^3^.

| **Bacterial Population** | **Forward Primer (5'-3')** | **Reverse Primer (5'-3')** |
| --- | --- | --- |
| *16s rRNA* (Universal) | CGTGCCAGCCGCGGTAATACG | GGGTTGCGCTCGTTGCGGGACTTAACCCAACAT |
| *Bifidobacterium* | GGGTGGTAATGCCGGATG | CCACCGTTACACCGGGAA |
| *Lactobacillus* | CATCCAGTGCAAACCTAAGAG | GATCCGCTTGCCTTCGCA |
| *Escherichia* | GACCTCGGTTTAGTTCACAGA | CACACGCTGACGCTGACCA |
| *Clostridium* | AAAGGAAGATTAATACCGCATAA | ATCTTGCGACCGTACTCCCC |

18S rRNA, 18S ribosomal subunit.

**Supplementary Material References**

1. Beasley, J. T. *et al.* Metabolic engineering of bread wheat improves grain iron concentration and bioavailability. *Plant Biotechnol. J.* 1–13 (2019). doi:10.1111/pbi.13074
2. Hou, T. & Tako, E. The in ovo feeding administration (*Gallus gallus*)—An emerging in vivo approach to assess bioactive compounds with potential nutritional benefits. *Nutrients* **10**, (2018).
3. Zhu, X. Y., Zhong, T., Pandya, Y. & Joerger, R. D. 16S rRNA-Based Analysis of Microbiota from the Cecum of Broiler Chickens. *Appl. Environ. Microbiol.* **68**, 124–137 (2002).
